# Weak Population Structure in European Roe Deer (*Capreolus capreolus*) and Evidence of Introgressive Hybridization with Siberian Roe Deer (*C. pygargus*) in Northeastern Poland

**DOI:** 10.1371/journal.pone.0109147

**Published:** 2014-10-01

**Authors:** Juanita Olano-Marin, Kamila Plis, Leif Sönnichsen, Tomasz Borowik, Magdalena Niedziałkowska, Bogumiła Jędrzejewska

**Affiliations:** 1 Mammal Research Institute, Polish Academy of Sciences, Białowieża, Poland; 2 Leibniz Institute for Zoo and Wildlife Research, Berlin, Germany; University of Florence, Italy

## Abstract

We investigated contemporary and historical influences on the pattern of genetic diversity of European roe deer (*Capreolus capreolus*). The study was conducted in northeastern Poland, a zone where vast areas of primeval forests are conserved and where the European roe deer was never driven to extinction. A total of 319 unique samples collected in three sampling areas were genotyped at 16 microsatellites and one fragment (610 bp) of mitochondrial DNA (mtDNA) control region. Genetic diversity was high, and a low degree of genetic differentiation among sampling areas was observed with both microsatellites and mtDNA. No evidence of genetic differentiation between roe deer inhabiting open fields and forested areas was found, indicating that the ability of the species to exploit these contrasting environments might be the result of its phenotypic plasticity. Half of the studied individuals carried an mtDNA haplotype that did not belong to *C. capreolus*, but to a related species that does not occur naturally in the area, the Siberian roe deer (*C. pygargus*). No differentiation between individuals with Siberian and European mtDNA haplotypes was detected at microsatellite loci. Introgression of mtDNA of Siberian roe deer into the genome of European roe deer has recently been detected in eastern Europe. Such introgression might be caused by human-mediated translocations of Siberian roe deer within the range of European roe deer or by natural hybridization between these species in the past.

## Introduction

Historical and recent events, shaped by both natural and anthropogenic factors, play an important role in the current patterns of genetic variation within the species. Climatic changes during the Quaternary, for example, defined the major genetic subdivisions of different taxa around the globe [1]. In more recent times, human practices such as agriculture, deforestation, development of infrastructure, hunting, and introduction of alien species, among others, have greatly affected the dynamics of natural populations, with consequences in the levels and distribution of genetic diversity within the species [2]–[4]. The microevolutionary consequences of human practices might have profound effects not only on threatened species living in small and isolated populations, but also on common and widespread species subject to strong management practices (e.g. ungulates) [5], [6].

The European roe deer (*Capreolus capreolus*), one of the most common ungulates in Europe and an important game species, is distributed across the European continent from the Mediterranean to Scandinavia. Major genetic subdivisions within the European roe deer are probably the result of historical vicariant events in southern glacial refugia [7]. More recently, the European roe deer experienced considerable reductions in population numbers or even local exterminations at several locations within its continuous range, mainly as a consequence of deforestation and over-hunting during the late 19^th^ and early 20^th^ centuries, [8]–[10]. Re-introduction and re-stocking programs (often using non-indigenous animals) were carried out in a number of places in the world, both with hunting and conservational purposes [8], [11]. Clearly, non-indigenous sources of individuals and local exterminations may have a tremendous impact on the genetic diversity of local populations of roe deer. In fact, genetic signatures (e.g. admixture, genetic drift) of such anthropogenic disturbances have been reported in several locations [7], [12]. Additionally, recent studies have documented the introgression of Siberian roe deer (*C. pygargus*) mitochondrial DNA (mtDNA) genes into European roe deer populations in Poland, Lithuania and Russia [13], [14]. Hybridization between these two closely related species might have been caused by natural processes which took place in the past or might be, at least partly, an effect of human-mediated introductions of Siberian roe deer within the range of European roe deer. Many events of such introductions took place in the European part of Russia and other countries in eastern Europe [8].

Human practices have not been completely detrimental for the European roe deer and, in fact, population expansions during the last two centuries have been attributed, among other factors, to the extension of cultivated fields providing a suitable and abundant source of food [9], [15]. The ability of the European roe deer to thrive in modern human-modified landscapes might be related to its considerable morphological, behavioral and ecological variability, and to its ability to exploit a great variety of habitats (e.g. broadleaved, coniferous and mixed forests, agricultural landscapes, ecotonal strips, lowlands and highlands) [9]. Morphological, behavioral and ecological differences among animals living in areas with contrasting levels of forest cover have been described [16]–[18]; accordingly, distinct field and forest ecotypes of the species have been recognized, even though there is no consensus about the validity of this distinction [19]. Moreover, genetic analyses at allozyme loci did not support the distinction of field and forest ecotypes within the species [20], and more powerful genetic markers (i.e. microsatellites) have not yet been used to investigate the differentiation between animals living in environments with contrasting levels of forest cover. The European roe deer has also served as a model species for investigating the genetic effects of fragmentation and human disturbance. It has been shown, for example, that the combination of several landscape features (i.e. highways, rivers, canals) may lead to population genetic differentiation [21], and that genetic discontinuities correlate with transportation infrastructure [22].

In this study we investigated the influence of contemporary (ecological) and past events on the patterns of genetic diversity and population differentiation of European roe deer in northeastern Poland. Unlike in most of west Europe, our study site comprises vast areas of conserved primeval forests where the European roe deer was never driven to extinction. As such, it provides an interesting comparison with previous genetic studies in central and western Europe, where the effects of recent drastic habitat fragmentation and reductions in population numbers are likely to have profound influences on the patterns of genetic diversity of the species. On the other hand, our study site is located within an area where introgression of mtDNA of Siberian roe deer into the genome of European roe deer has recently been described [13], [14]. Interestingly, local reports from the late 19^th^ and early 20^th^ centuries document the introduction of non-indigenous Siberian roe deer within our study site, with the purpose of increasing the size and quality of hunting trophies [23]. We analyzed molecular genetic variation at both mitochondrial and nuclear DNA across three sampling areas. The pattern of genetic structure was investigated with respect to the amount of forest cover, in order to establish whether modern and powerful genetic markers support the distinction between field and forest roe deer. The pattern of mtDNA diversity was analyzed in the context of previous phylogeographical studies of roe deer and historical literature.

## Materials and Methods

### Ethics statement

Government approval or licenses were not required for the collection of tissue samples (i.e. skin or muscles) from legally hunted animals, which were obtained through hunters and hunting associations. Hunted animals were shot with rifle during the hunting season, following the rules of the Polish hunting law. No animals were killed specifically for this study. Permissions for sampling of live animals were obtained from the Polish Ministry of Environment (Permit No. DLOPik-L-gl-6713/86b/07/ab) and the Local Ethical Commission in Białystok (Resolution No. 46/2008).

### Study site and sampling of European roe deer

**Figure 1:**
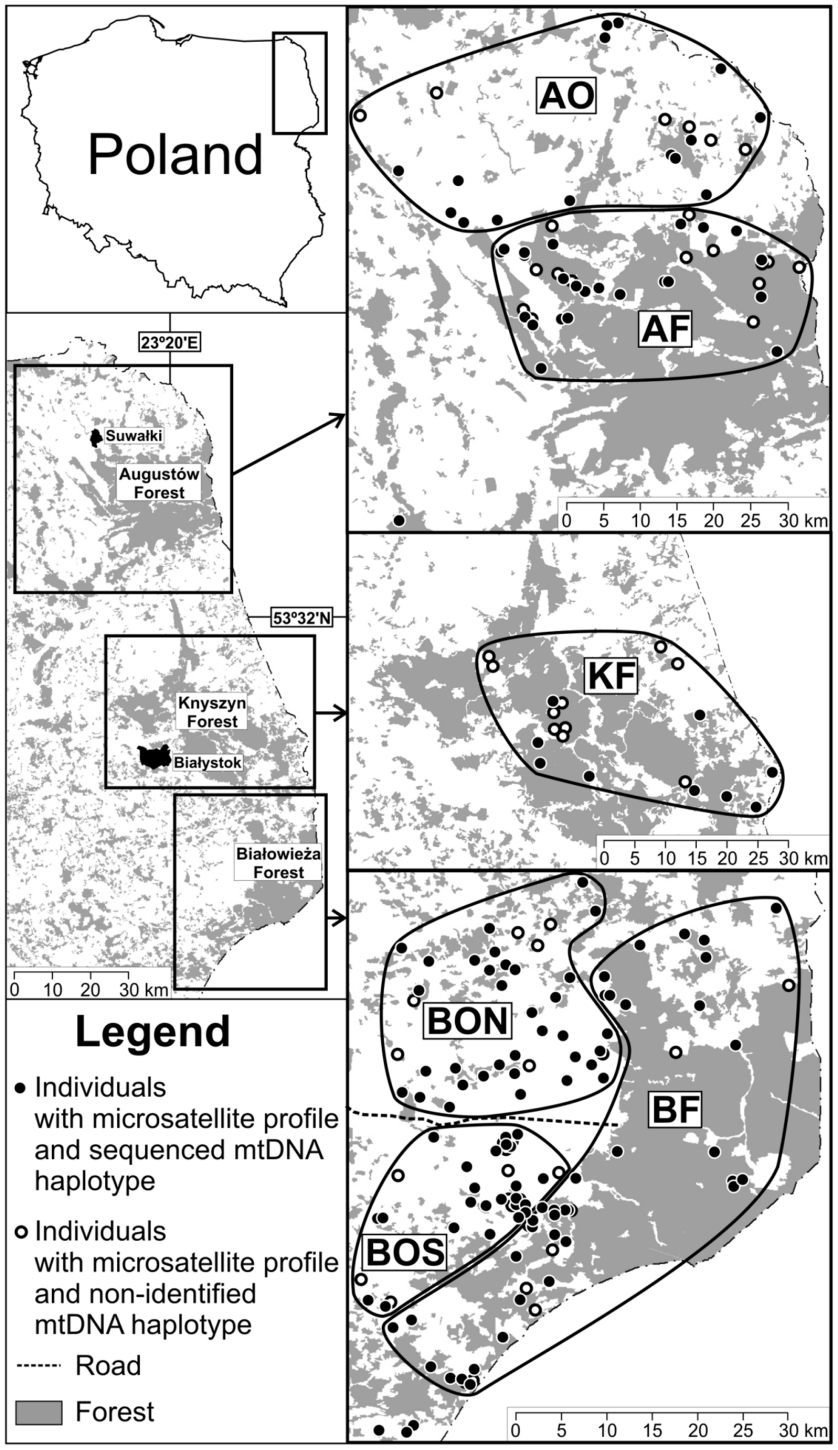
Study area and roe deer sampling in northeastern Poland. Samples were collected in and around three lowland forests: Augustów (A), Knyszyn (K) and Białowieża (B). Filled symbols indicate samples that were genotyped at both microsatellites and mtDNA control region, whereas open symbols indicate samples that were only typed with microsatellites. Polygons were drawn around regions with contrasting degrees of forest cover within each sampling site, which were used to define field (O - open habitat) and forest (F) groups of roe deer; a further subdivision in Białowieża separates open habitats in the north (N) and south (S) of a main road.

The study area consisted of three sampling sites (Białowieża, Knyszyn and Augustów, ca. 5340 km^2^), distributed latitudinally in northeastern Poland (22°33′ –22°53′E, 52°26′ –54°17′N; max. span in distance: N–S 200 km, E–W 107 km; Fig. 1). The landscape in all sampling sites can be divided into three distinctive categories: open (arable lands covered by crop plantations and meadows), closed (coniferous, deciduous, and mixed forests), and mosaics of the arable land, meadows and forests. The northernmost sampling site, Augustów, covers ca. 2750 km^2^ (with 58% of open and 33% of forested areas, with most of the latter belonging to Augustów Forest). The second sampling site, Knyszyn (740 km^2^, with 71% of forests mainly belonging to Knyszyn Forest, and 28% of open areas), is located ca. 92 km south of Augustów. The southernmost sampling site, Białowieża (1850 km^2^, with 44% of forests, mainly belonging to Białowieża Primeval Forest, and 53% of open land), is located ca. 56 km from Knyszyn.

We used a total of 328 roe deer tissue samples collected between 2004–2011∶234 in Białowieża, 22 in Knyszyn, and 72 in Augustów (Fig. 1). With the exception of 33 live-captured individuals used for telemetry studies [24] and 4 lynx preys, samples were obtained through hunters and hunting associations, and consisted of parts of skin or muscles from legally hunted animals. The geographic coordinates of the samples were assigned according to the information provided by the hunters and were defined as the geometric central point of a hunting district where a particular animal was hunted. In case of the individuals followed by telemetry, the geographic coordinates corresponded to the place of capture. All samples were stored in 96% ethanol at −20°C prior to DNA extraction.

### Definition of ecologically-relevant groups of roe deer

In order to investigate the influence of the amount of forest cover on the pattern of genetic structure, we defined groups of European roe deer inhabiting contrasting environments within our three sampling sites. We visually inspected the distribution of forest cover within the sampling sites with ArcMap 9.3.1 (ESRI Inc. 2009) (Fig. 1); individuals within areas of continuous forest cover were grouped as forest roe deer, while individuals within areas of open or mixed habitats were grouped as field roe deer. Given the potential role of road infrastructure on the genetic structuring of roe deer [22], field roe deer from the Białowieża area were further subdivided in two subgroups, one north and one south of the main road in that region. Groups were delimited by complex polygons and the percentage of forest cover within these polygons was calculated. Four samples could not be assigned to groups due to their relatively large geographic isolation with respect to the other samples.

### Genotyping

Total DNA from all samples was extracted with the Qiagen DNeasy Blood and Tissue Kit following the manufacturer’s protocols. We genotyped each individual with 16 microsatellite markers that were reported as polymorphic for roe deer (Table S1). Products were separated in an ABI 3130 xl Genetic Analyzer with the GeneScan 400HD ROX Size Standard (Applied Biosystems). Genotypes were read with the software GeneMarker (Softgenetics). A fragment of mtDNA control region was amplified by PCR with the primers L-Pro and H-Phe [25]. Cycling conditions were 95°C for 15 min; 35 cycles of 94°C for 15 s, 56°C for 15 s, and 72°C for 1 min; and 72°C for 10 min. PCR products were purified using Clean Up (A&A Biotechnology, Gdańsk, Poland). Sequencing reactions were carried out in a 10 µl volume using the Big Dye sequencing kit v.3.1 (Applied Biosystems) with the forward primer. Products were purified with the Exterminator kit (A&A Biotechnology) and separated on an ABI 3130 xl Genetic Analyzer (Applied Biosystems). Sequencing results were analyzed with the ABI DNA Sequencing Analysis software and aligned in BioEdit v.7.0.9 [26].

### Analyses of microsatellite data

We checked for duplicated samples with the package *allelematch* [27] for R [28]. From the 328 genotyped samples, 9 were excluded from further analysis because they showed identical or almost identical (>93% of similarity) profiles to other samples (i.e. some individuals were sampled more than once), or due to failures in amplification at 4 or more microsatellite loci. Allele frequencies, observed and expected heterozygosity, deviations from Hardy–Weinberg equilibrium (HWE), and F_IS_ were calculated with GENEPOP 4.1.4 [29] for each sampling area. Allelic richness, a measure that accounts for differences in samples sizes in estimates of the number of alleles, was calculated with HP-RARE 1.0 [30]. Null allele frequencies and genotyping errors were estimated with the program Micro-Checker [31]. False Discovery Rate corrections [32] were performed in R [28] to account for multiple testing.

In order to account for the possibility of genetic changes occurring over time, we performed an analysis of molecular variance (AMOVA) in Arlequin v.3.5.1.3 [33] with the samples grouped into year classes within each sampling area. We investigated the occurrence of population structure and the pattern of genetic differentiation among field and forest roe deer with different methods. First, we used the clustering program STRUCTURE [34]. We chose the admixture model and the option of correlated allele frequencies between populations, with and without sample location and the grouping of field and forest roe deer as prior information [35]; we let the parameter alpha (the degree of admixture between subpopulations) be inferred from the data and set lambda (the allele frequency prior) to 1. We conducted 10 independent replicate analyses for values of K (number of genetic clusters) between 1 and 10 with a burn-in period of 10 000 iterations and 100 000 Markov chain Monte Carlo (MCMC) cycles. We used the STRUCTURE HARVESTER [36] to compile and visualize the results from the STRUCTURE runs and to calculate ΔK [37]. Second, we used the spatial model of clustering implemented in the program GENELAND [38]. Uncertainty in the coordinates was set to 8×10^−3^ to account for ca. 1 km of uncertainty in the location assigned to the samples [21], and the maximum number of populations was set to 10. We used the correlated allele frequency model as described in Guillot [38], for runs with 1×10^6^ MCMC iterations. Ten independent runs were made to look for convergence in the number of estimated K. Third, we further investigated the genetic substructure, isolation by distance, and differentiation groups of field and forest roe deer by means of multivariate methods implemented in the R-package *adegenet* [39]. An advantage of multivariate methods over other clustering algorithms is that the former do not rely on Hardy-Weinberg and linkage equilibrium for summarizing genetic variability. We used spatial Principal Component Analysis (sPCA) [40] in order to investigate spatial genetic patterns within the study area; the connection network between individuals was defined with the Delaunay triangulation [41], and global and local tests (with 9999 permutations) were performed as an aid for selecting the structures to be interpreted. Discriminant Analysis of Principal Components (DAPC) [42], a method designed to identify and describe clusters of genetically similar individuals, was used to visualize the genetic relatedness/differentiation between the previously defined groups of field and forest roe deer. For this DAPC, forty principal components of PCA and all (5) discriminant functions were retained. The function *find.clusters* was used to identify the number of clusters (K) in our data and to compare with the prior groups; for this, we covered values of K between 1 and 10, and followed the procedure outlined in Jombart *et al*. [42]. Fourth, we performed an analysis of molecular variance (AMOVA) in Arlequin v.3.5.1.3 [33] with the field and forest groups nested within each sampling area, for a hierarchical partition of the genetic variance within and among sampling sites and groups. Finally, we calculated D_Jost_ [43] as a measure of differentiation between field and forest groups and sampling areas with the R-package *diveRsity* v.1.3.2 [44]. We investigated the pattern of nuclear genetic differentiation among the mtDNA clades found, as well as the influence of individuals with Siberian haplotypes (see Results) on the genetic structure of roe deer in the study area by performing additional STRUCTURE analyses and DAPC; for this, prior groups were defined according to the mtDNA clades or samples with Siberian haplotypes were excluded from the analyses.

### Analyses of mtDNA data

We obtained good-quality mitochondrial control region sequences (ca. 610 bp) for 241 out of the 319 unique samples. For the remaining 78 samples, repeated amplification attempts with different PCR and sequencing conditions did not result in readable sequences. Mitochondrial control region sequences were aligned against a reference sequence of European roe deer (GenBank accession number AY625869.1) [7] and manually edited in BioEdit v.7.0.5.3 [26]. Measures of genetic diversity were estimated with Arlequin v.3.5.1.3 [33]. We determined the position of our roe deer haplotypes within the phylogenetic clades of the species described by Randi *et al*. [7], using published European and Siberian roe deer mtDNA sequences [7], [13], [14], [45]–[51]. As the recently published mtDNA sequences by Matosiuk *et al*. [14] were shorter than ours, we performed the phylogenetic reconstruction both with our full-length sequences (610 bp) and with shortened ones (510 bp). Phylogenetic trees were reconstructed using MEGA 5 [52], with the neighbour-joining procedure and Tamura and Nei’s TN93 genetic distance model, as described by Randi *et al*. [7]. Support for the internodes was assessed after 10 000 bootstrap resampling steps. We inferred the haplotype genealogy of our samples with a mtDNA network constructed with the median-joining procedure in Network 4.6 (http://www.fluxus-engineering.com/). Genetic differentiation among sampling sites and prior groups was estimated with AMOVA, F_ST_ [33] and spatial analysis of molecular variance (SAMOVA) [53], with field and forest groups of roe deer nested within each sampling area. In order to investigate the influence of individuals with Siberian haplotypes (see Results) on the pattern of genetic diversity/differentiation, we repeated all the analyses with samples carrying European haplotypes only.

## Results

### Genetic diversity of European roe deer in northeastern Poland

The genetic diversity of roe deer at the three sampling sites in northeastern Poland was high, both at microsatellite and mitochondrial markers (Table 1). One microsatellite locus, BMS119, was monomorphic and therefore excluded from further analyses. The number of alleles at the remaining 15 microsatellite loci ranged between 2–14 (8.67 on average), and the mean observed heterozygosity was 0.60. None of the loci showed evidence of scoring errors due to large allele drop-out or stutter peaks. Significant deviations from HWE were detected in loci NVHRT21 and Roe1 (in Białowieża), NVHRT73 (in Białowieża and Augustów), and NVHRT71 and ETH225 (in all sampling sites); with the exception of locus Roe1, all the deviations from HWE were due to heterozygote deficit. The frequency of null alleles at the loci with significant deficit of heterozygotes ranged between 0.11–0.41, with the highest values for locus NVHRT71. F_IS_ measured across loci was positive in all sampling areas, although low (≤0.07). Due to the strong influence of locus NVHRT71 on results of the DAPC and estimates of population differentiation, and to its high frequency of null alleles, we excluded it and present the results of all analyses done with the remaining 14 loci.

**Table 1:** Microsatellite and mtDNA diversity of roe deer *Capreolus capreolus* at three sampling regions in northeastern Poland.

Parameter	Sampling site			Total
	Białowieża	Knyszyn	Augustów	
*Microsatellites*				
Sample size	230	20	69	319
No. of alleles/locus	8.33	5.87	7.07	8.67
Allelic richness	6.07	5.87	5.98	-
Private allelic richness	0.54	0.40	0.60	-
Observed heterozygosity	0.62*	0.58*	0.61*	0.61*
Expected heterozygosity	0.66	0.63	0.65	0.66
Fis	0.07	0.07	0.06	0.07
*mtDNA (all sampled roe deer)*				
Sample size	184	10	47	241
No. haplotypes	13	6	6	13
No. polymorphic sites	39 (6.4%)	34 (5.6%)	38 (6.2%)	39 (6.4%)
(% of sequence length)				
Haplotype diversity *h* (SD)	0.714 (0.029)	0.844 (0.103)	0.794 (0.030)	0.756 (0.025)
Nucleotide diversity π (SD)	0.024 (0.012)	0.026 (0.014)	0.025 (0.013)	0.025 (0.012)
Pairwise divergence *k* (SD)	14.457 (6.503)	15.844 (7.672)	15.547 (7.055)	14.994 (6.726)
*mtDNA (European roe deer only)*				
Sample size	84	4	31	122
No. haplotypes	10	3	5	10
No. polymorphic sites	24 (3.9%)	12 (2.0%)	20 (3.3%)	24 (3.9%)
(% of sequence length)				
Haplotype diversity *h* (SD)	0.766 (0.040)	0.833 (0.222)	0.778 (0.035)	0.848 (0.019)
Nucleotide diversity π (SD)	0.006 (0.003)	0.011 (0.007)	0.012 (0.006)	0.009 (0.005)
Pairwise divergence *k* (SD)	3.750 (1.910)	6.500 (3.817)	7.307 (3.506)	5.266 (2.561)

Microsatellite diversity is based on 15 polymorphic loci. MtDNA diversity is based on 610 bp control region sequence. SD: standard deviation; * significant deviation (<0.05) from Hardy-Weinberg equilibrium.

**Figure 2:**
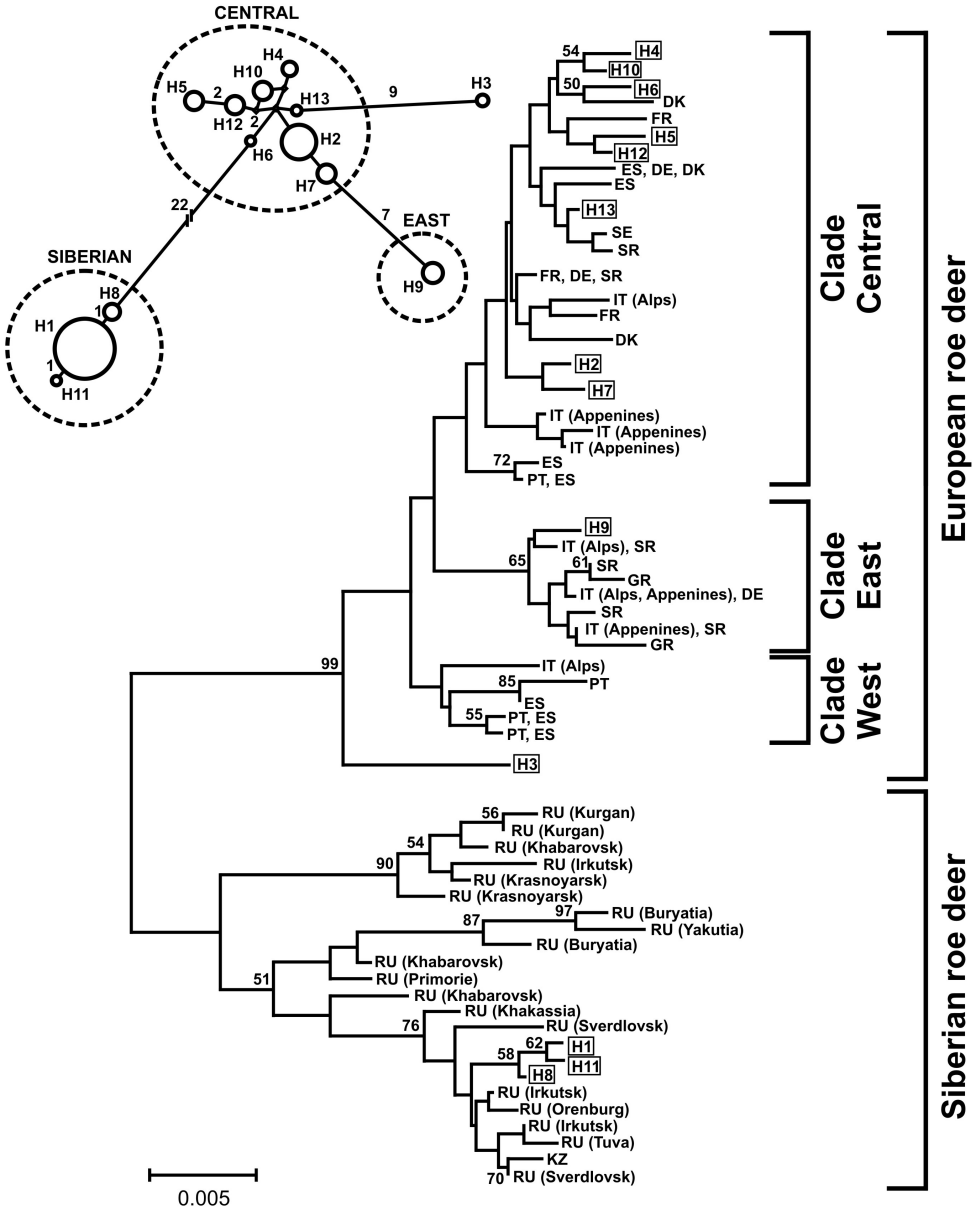
Phylogenetic relationship between the mtDNA haplotypes found in this study (H1–H13) and other published mtDNA control region sequences of European[7] and Siberian [45] roe deer. Geographic locations of published sequences of European roe deer (DE = Germany; DK = Denmark; ES = Spain; FR = France; GR = Greece; IT = Italy; PT = Portugal; SE = Sweden; SR = Serbia, Montenegro, and Kosovo) and Siberian roe deer (KZ = Kazakhstan; RU = Russia) are indicated in the phylogenetic tree. Numbers at nodes show support (≥50%) from 10 000 bootstrap replicates. European roe deer clades are defined according to Randi *et al*. [7]. In the median-joining network of the mtDNA haplotypes found in this study (upper-left), clades are grouped within punctuated circles. The size of the solid circles is proportional to the number of individuals with a given haplotype in the whole study area. Numbers above the branches indicate the mutation steps between two haplotypes.

A total of 13 different mtDNA control region haplotypes were defined by 39 polymorphic sites (38 substitutions, 1 deletion). Complete sequences have been deposited in GenBank with accession numbers KM068160–KM068172. The genetic diversity was high (Table 1). The phylogenetic and network analyses (Fig. 2, Fig. S1 and S2) revealed three haplotypes (H1, H8 and H11) that were highly divergent and grouped with Siberian roe deer. Surprisingly, these haplotypes were carried by more than half (50.6%) of the individuals sampled. Siberian haplotypes clearly increased the measures of mtDNA diversity in all sampling areas (Table 1). The remaining haplotypes corresponded to European roe deer; most of them (H2, H4, H5, H6, H7, H10, H12, H13) grouped within the Central clade described by Randi *et al*. [7], one (H9) grouped within the East clade, and one considerably divergent haplotype (H3) was included within the Central or Western clades of the species (Fig. 2, Fig. S1 and S2). The main topology of the trees including full-length and shortened sequences was similar (Fig. 2, Fig. S1 and S2). Numbers of the different haplotype-clades varied considerably across the study area. The proportion of individuals carrying haplotypes of Siberian roe deer declined northwards, from 55% in Białowieża and Knyszyn (pooled) to 34% in Augustów; the opposite trend was revealed in the frequencies of the European roe deer haplotype belonging to the clade East (2% in Białowieża-Knyszyn, 21% in Augustów) and haplotype H3 (0.5 and 8.5%, respectively).

### Genetic population structure

The visual inspection of the distribution and amount of forest cover within the study site resulted in the definition of 6 groups of roe deer (Fig. 1): 3 in forested areas (one in each sampling site, with 69 samples in Białowieża, 20 in Knyszyn, and 39 in Augustów) and 3 in more open fields (two in Białowieża: with 103 and 55 samples at the north and south of the main road, respectively; and one in Augustów with 29 individuals); 4 samples were not classified due to their geographic isolation. Field and forest groups clearly differed in their mean forest cover (17% vs. 70% for open and forested areas, respectively).

**Figure 3:**
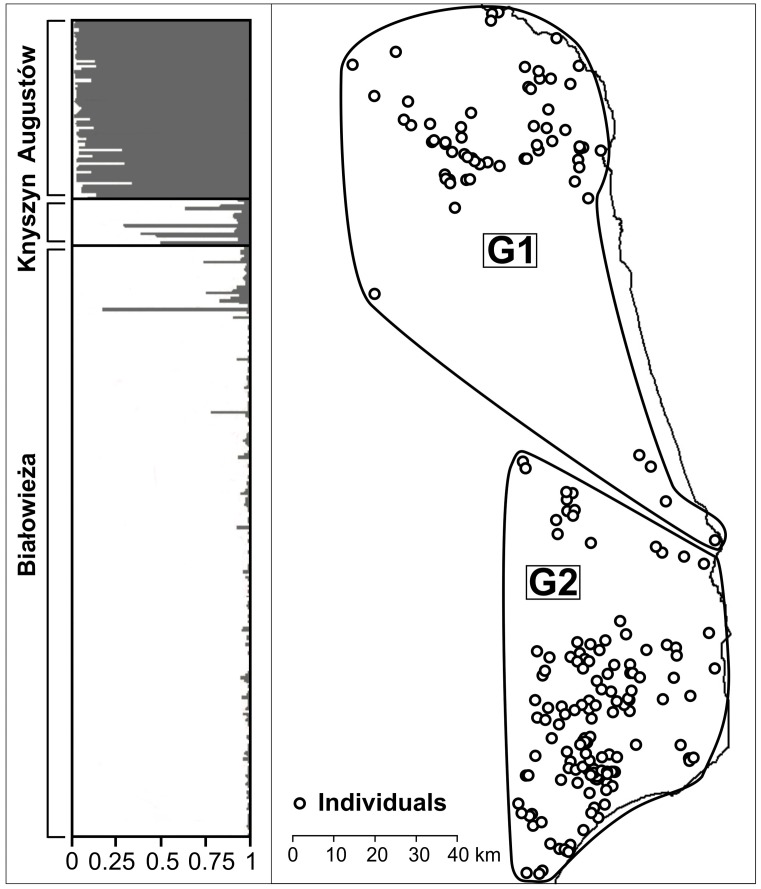
Population structure of roe deer in northeastern Poland according to microsatellite markers. Left panel: Results from the STRUCTURE analysis. The best supported number of genetic clusters was 2. Each vertical line represents one individual partitioned into 2 colored segments; each colored segment represents the estimated membership coefficient of the individuals to the two inferred clusters. Right panel: Map of estimated membership from the GENELAND analysis for K = 2. Each dot represents one individual and the polygons encircle individuals belonging to the two inferred genetic groups (G1, G2).

We found no evidence of genetic changes in the roe deer population over the study years (only 0.05% of the genetic variance at microsatellite loci occurred among years, F_SC_ = 0.0005, p = 0.3216). The clustering analyses in STRUCTURE (both with and without spatial prior information) showed a peak in the mean posterior probability (Ln P(D)) and in ΔK for K = 2. The pattern of assignment of the individuals to the two inferred clusters divided the study area along the north-south axis (Fig. 3 left panel): individuals from Białowieża (in the south) were mostly assigned to one cluster, whereas individuals from Augustów (in the north) were mostly assigned to the second cluster; individuals from Knyszyn (located between Białowieża and Augustów), on the other hand, showed a mixed pattern of assignment to the two inferred clusters. The GENELAND analysis (which employs a spatially-explicit clustering approach) also returned K = 2 as the most likely number of genetic clusters in six out of ten independent runs. The results matched exactly the pattern observed with STRUCTURE: all individuals from Białowieża and Augustów were assigned to either one or the other cluster, whereas the individuals from Knyszyn were assigned to both (Fig. 3 right panel). Neither the STRUCTURE nor the GENELAND analyses suggested a further population subdivision that could reflect genetic differences between individuals assigned to field and forest groups.

**Figure 4:**
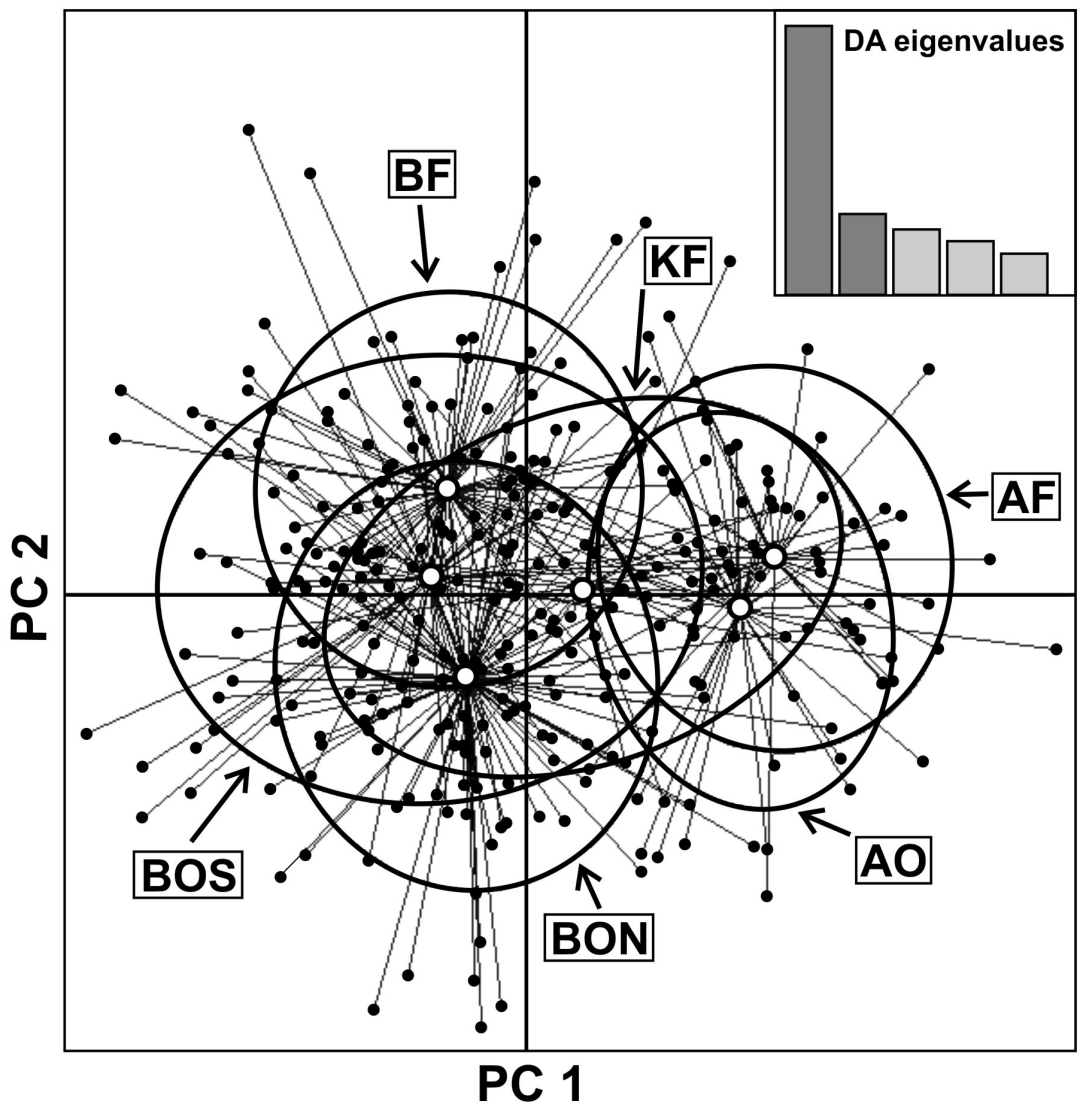
Nuclear genetic differentiation of roe deer groups inhabiting fields and forests according to the Discriminant Analysis of Principal Component (DAPC). The first two principal components are shown. Denotations of groups of roe deer as in Fig. 1.

Similar results were obtained with the multivariate analyses. The first positive eigenvalue or global score of the sPCA was retained based on its spatial and variance components. A global test confirmed the existence of a global structure in our data (i.e. positive spatial autocorrelation, max(t) = 0.0104, *p* = 0.0001), whereas the local test did not detect any local pattern (i.e. negative spatial autocorrelation, max(t) = 0.0045, non-significant). The first global score differentiated Białowieża from the other two sampling areas, which appeared similar to each other (not shown). The slight pattern of isolation by distance, on the other hand, was not significant. The first principal component of the DAPC separated the individuals from Białowieża and Augustów in the extremes, sharing the space with the individuals from Knyszyn, which were placed in the middle between the other two sampling sites; a high degree of overlap between field and forest groups within sampling areas, and between animals on either side of the road in Białowieża was evident (Fig. 4). The function *find.clusters* identified 3–5 groups in the data; these groups did not match the prior groups defined by sampling areas and forest cover, and their members did not show any spatial clustering. The AMOVA with forest and field roe deer grouped within sampling sites revealed that 98.5% of the genetic variance occurred within the groups, whereas only 0.3% of the variance occurred among groups within sampling areas, and 1% among the three sampling areas. The pairwise measures of differentiation (D_Jost_) between groups of samples were extremely small (<0.028, in a 0–1 scale where 0 indicates no differentiation and 1 reflects complete differentiation, Table 2). The pattern of nuclear genetic structure did not change when only individuals with European mtDNA haplotypes were considered.

**Table 2:** Pairwise measures of differentiation (D_Jost_) and Fst between sampling sites and groups of roe deer inhabiting areas with different degree of forest cover (denotations of groups as in Fig. 1).

Group	BOS	BON	BF	KF	AO	AF
BOS	–	0.0002	0.0000	0.0051	0.0133	0.0180
BON	0.0037	–	0.0042	0.0053	0.0234	0.0133
BF	−0.0017	0.0604*	–	0.0032	0.0278	0.0179
KF	−0.0471	−0.0473	0.0016	–	0.0028	0.0036
AO	0.0996*	0.1981*	0.0507	0.1075	–	0.0011
AF	0.0127	0.0790*	0.0091	−0.0018	0.0136	–

Above diagonal: D_Jost_ calculated with microsatellite data; below diagonal: Fst calculated with mtDNA sequence data. The 95% confidence interval of all D_Jost_ estimates ranged between 0.0001–0.1171. * Significant (<0.05) Fst values.

**Figure 5:**
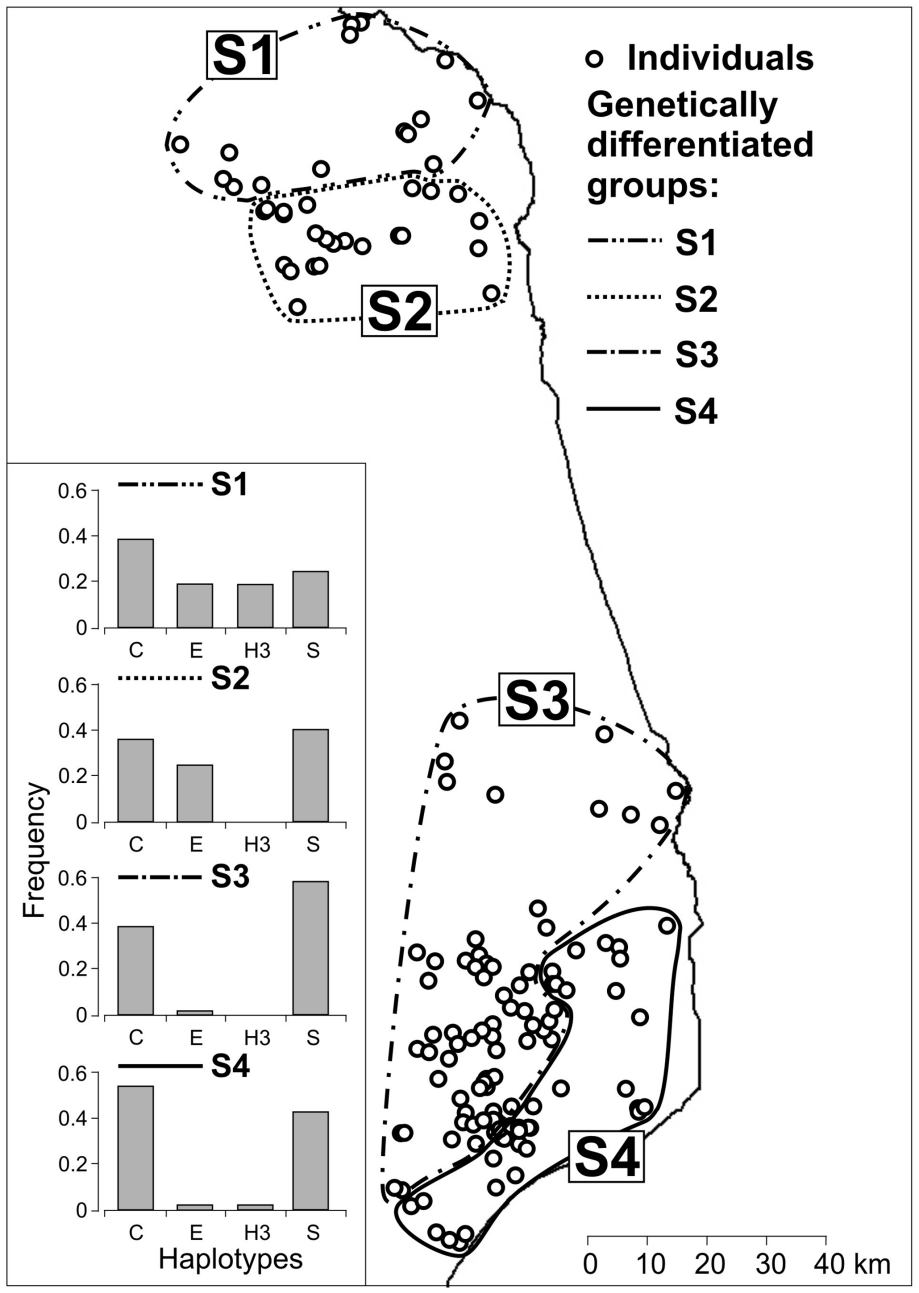
Groups of roe deer (S1–S4) based on mtDNA data according to SAMOVA, and frequencies of haplotype clades (C = Central, E = East, H3, S = Siberian) in each of the defined groups.

The mtDNA data revealed a similar pattern of genetic structure. Białowieża and Augustów showed some degree of differentiation reflected in relatively large and significant F_ST_ values (Table 2); F_ST_ values among roe deer ecotypes within the sampling areas, on the other hand, were mostly small and non-significant (Table 2). The AMOVA performed with mtDNA data, with forest and field roe deer grouped within sampling sites, showed that 93.2% of the genetic variance occurred within groups, 2.4% among groups within sampling areas, and 4.3% among the sampling sites. According to the SAMOVA (Fig. 5), the roe deer samples could be divided into 4 regional groups: two separating forest and field areas in Augustów, one grouping Knyszyn forest and field areas of Białowieża, and the last one corresponding to Białowieża forest; this subdivision, however, was not further supported by F_ST_ measures of genetic differentiation (Table 2).

### Nuclear genetic differentiation among individuals with Siberian and European mtDNA haplotypes

**Figure 6:**
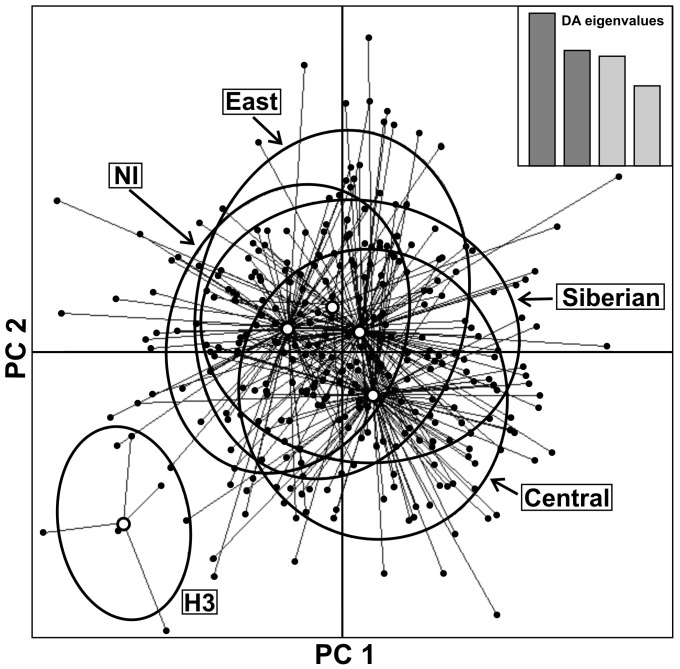
Scatterplot of the DAPC showing the nuclear genetic differentiation (according to microsatellite markers) between groups of individuals with European (Central, East and H3), Siberian and non-identified (NI) mtDNA haplotypes. The first two principal components are shown.

The measures of microsatellite diversity did not differ among groups of animals with European, Siberian and non-identified mtDNA haplotypes (not shown). The STRUCTURE analysis and DAPC with groups defined according to the mtDNA clade of the individuals (i.e. Siberian, clade Central, clade East, H3 or non-identified) suggested a genetic homogeneity in the nuclear genetic composition among most clades. In the DAPC, individuals with the haplotype H3 grouped apart from all the other individuals with different haplotypes (Fig. 6).

## Discussion

### Genetic diversity of roe deer in northeastern Poland in historical context

The relatively high levels of variability and the slight heterozygote deficiency at microsatellite loci of roe deer in northeastern Poland are consistent with findings from previous studies conducted across Europe (e.g., [54], [55]). The high frequency of null alleles at loci with significant deficit of heterozygotes indicates a plausible cause for the observed departures from HWE. Other causes of heterozygote deficit (i.e. sex-biased dispersal, yearly shifts in allele frequencies, inbreeding), although cannot be completely ruled out, are not supported by other roe deer studies [56] and our own observations.

The values of mtDNA diversity indicate a high effective population size of roe deer in northeastern Poland, and are likely to reflect the complex history of the species in this region. Almost half of the individuals carried European roe deer mtDNA haplotypes. From these, eight were closely related and belonged to the Central clade [7], which, according to the mtDNA network and given that the roe deer was never extinct in northeastern Poland [57], are likely to be native in this region. The two remaining European haplotypes (H9 and H3), on the other hand, were highly divergent and were represented by few individuals mostly found in the north of the study area. Haplotype H3 was previously described by Zvychainaya *et al.* [45] in a European roe deer population in western Russia. In our analyses, this haplotype clustered within the Western or the Central European roe deer clades and, therefore, its origin cannot be clearly determined. Four of the European roe deer haplotypes found in our study (H4, H5, H6 and H12) have not been previously described by other authors [7], [13], [14], [45]–[51].

More than half of all the individuals studied carried Siberian roe deer mtDNA haplotypes, which is in concordance with the results of recently published studies of Lorenzini *et al.* [13] and Matosiuk *et al.* [14], who conducted phylogeographic analyses of roe deer populations including samples from Poland and Lithuania. One out of the three Siberian mtDNA haplotypes found in our study areas (H11) was not described before by other authors [7], [13], [14], [45]–[51]. In our study, individuals possessing Siberian roe deer haplotypes did not show any remarkable phenotypic feature that would put in doubt their identity as European roe deer (hunting associations, pers. comm., and our own observations), nor did they present difficulties for amplification of nuclear markers. Moreover, no nuclear genetic differentiation (including sex-specific markers) (see [14]) among animals carrying divergent mtDNA haplotypes was found.

Phylogeographic studies of the genus *Capreolus* estimate a divergence time between European and Siberian roe deer of about 2–3 million years [25]. Nowadays, their natural distribution is allopatric, with European roe deer occupying most of western Europe, and Siberian roe deer naturally found across the temperate zone of eastern Europe and Asia. A narrow contact zone between the two species occurs at the westernmost limit of the Siberian roe deer distribution, at the Khoper and Don rivers in the European part of Russia [58]. European and Siberian roe deer differ in characters such as body size, morphometric traits and karyotype [8]. Despite a large degree of reproductive isolation between the two species, hybridization in captivity has been demonstrated and it probably also occurs in natural conditions but is difficult to document [8]. Successful production of hybrids is more likely to occur in crosses between Siberian females and European males, as the smaller European roe deer females usually die while giving birth to large hybrid fetuses or give birth to dead young [8], [58]. Introgressive hybridization of Siberian mtDNA into the European roe deer gene pool is, therefore, not unlikely. However, it has not been reported in phylogeographic studies of European roe deer at central and western parts of its geographical distribution [7], [12], [54], [59] and was only recorded in the eastern part of the species range (this study, [13], [14]), reaching up to 78% of hybrids in a roe deer population in the Moscow region, Russia [46].

Given the fact that our study area lies far beyond the actual range of Siberian roe deer [8], the finding of a high proportion (but low numbers) of Siberian haplotypes in otherwise European-looking roe deer in northeastern Poland is particularly interesting. Both anthropogenic factors and natural processes have an impact on the present distribution of roe deer in Europe [8]. On one hand, our observations might be caused by human-mediated introduction(s) of Siberian roe deer [8], [23] and the posterior introgressive hybridization with the local European roe deer; on the other hand, they might be an effect of natural processes which took place in eastern Europe in the past (as postulated by [13] and [14]). In fact, the structure of the mtDNA network of the Siberian haplotypes found in this study (with one very common haplotype and two minor ones) might indicate a bottleneck caused by a low number of founders. Historical evidence from the Białowieża region supports the hypothesis of human-mediated introductions, with a first recorded translocation and posterior release of eight Siberian roe deer (probably from the Ural mountains) and their progeny to the Białowieża Primeval Forest (BPF) in 1891 [23]. At that time, the population of European roe deer in BPF was estimated at about 600 individuals, and since then its numbers fluctuated between 300 and 6100 animals [57]. Posterior translocations from BPF to other areas in Poland were also described by Karcov [23]. Unfortunately, not all translocations were documented in written sources, but many of them might have taken place in eastern Poland (personal communication with members of the Polish Hunting Association). The fact that the number of individuals carrying haplotypes of Siberian roe deer declined northwards from Białowieża (where the documented introductions took place) to Augustów Forests, also supports the hypothesis of human-mediated translocations.

Alternatively, the presence of mtDNA of Siberian roe deer in European roe deer populations in eastern Europe might be caused by sympatric distribution of the two species in the past and natural introgression of Siberian roe deer mtDNA into the European roe deer genome [13], [14]. According to Danilkin [8], the historical range of Siberian roe deer spread further west than the present distribution of the species. Lorenzini *et al.* [13] and Matosiuk *et al.* [14] suggest that, after the Last Glacial Maximum, the range of Siberian and European roe deer overlapped in central and eastern Europe, and natural hybridization occurred at that time. Interestingly, Siberian haplotypes found both in our study and by Matosiuk *et al.* [14] grouped in the phylogenetic tree with samples collected in Kazakhstan, the Irkutsk region and Khakassia, which might suggest that founders of European roe deer populations in northeastern Poland originated from this area. Lorenzini *et al.* [13] claims that the lack of divergence in nuclear DNA between individuals with Siberian and European mtDNA can be a proof that the mtDNA introgression is much older than 200 years ago, when the translocation by humans took place. Given the lack of genetic data from roe deer populations in large areas of eastern Europe and western Asia, it is not possible, in our opinion, to definitively establish whether the introgression of Siberian roe deer genes into the local populations of European roe deer has natural or human-mediated causes. The genetic analyses of ancient samples of roe deer from eastern Europe could help to resolve this issue.

Introgression of Siberian roe deer mtDNA into local populations of European roe deer may have evolutionary implications. Mitochondrial DNA can be responsible for adaptation of organisms to changing environmental conditions (e.g. [60], [61]). In our case, hybrids possessing Siberian mtDNA could be better adapted, for example, to severe winters, an important agent of roe deer mortality in eastern Poland [62]. A similar conclusion was drawn by Matosiuk *et al.* [14], who compared the distribution of Siberian roe deer haplotypes in the population of European roe deer in Poland with environmental factors. However, the hypothesis of adaptive advantage of Siberian roe deer gene introgression into European populations calls for further studies.

### Population structure and landscape features

All of the microsatellite and mtDNA analyses showed a pattern of slight north-south differentiation that corresponded to the geographic origin of the roe deer samples. The continuous distribution of roe deer across the whole study area, the lack of significant barriers for their movement, the low differentiation between sampling sites, and the absence of a significant pattern of isolation by distance suggests that populations from Augustów and Białowieża should be seen as extremes in the continuous genetic variation of roe deer within the study site, rather than as discrete differentiated groups. Given that the mean dispersal distances of roe deer are small (less than 2 km) [9], and although the furthest recorded distance from the place of capture in our study area was relatively large (22.1 km), it is not surprising to find a certain amount of differentiation between the furthermost areas (separated by ca. 140 km) in this study. These low levels of structure among sampling areas were not only caused by individuals carrying Siberian haplotypes, as analyses excluding these individuals revealed the same general pattern found with all samples. The lack of genetic differentiation among groups of animals found on different sides of the main road in the Białowieża area, suggests that this type of non-fenced transportation infrastructure does not act as a barrier for roe deer dispersal. Fenced highways, in contrast to non-fenced roads, railways and other linear landscape elements, seem to have an impact on the movement of roe deer and cause some degree of population differentiation [22]. The pattern of nuclear genetic differentiation in the studied roe deer population did not considerably differ from the one found with mtDNA, suggesting that both males and females have a similar contribution to the observed pattern. Female philopatry in the roe deer might result in a stronger signal of genetic differentiation at mtDNA (relative to nuclear markers) at relatively large spatial scales [63]. At smaller spatial scales, however, the lack of differences in fine-scale genetic structuring between males and females [64] might explain the concordant pattern of mitochondrial and nuclear genetic structure found here.

We did not find a clear genetic support for the subdivision of roe deer into field and forest ecotypes. The genetic differentiation between groups of individuals inhabiting areas with different levels of forest cover within the same sampling site was very small; this was true at all three sampling areas and with both nuclear and mitochondrial markers. Our results with presumably neutral genetic markers support earlier findings of a lack of differentiation between roe deer ecotypes using allozymes [20]. These results are not surprising; genetic differentiation between roe deer ecotypes may only occur through a mechanism (e.g. spatial, behavioral, morphological or ecological) that could generate some kind of reproductive isolation between them. In all three sampling regions, there were no evident barriers for dispersal between areas with different degrees of forest cover. In fact, with telemetry data, we were able to document the dispersal of individuals between habitats with different degrees of forest cover (L. Sönnichsen, unpublished data). The slight morphological, physiological and behavioral differences between roe deer inhabiting forests and fields that have been used to support the definition of ecotypes [16]–[18], are thus likely to reflect the phenotypic plasticity of the species in response to differences in forest cover, predation pressure and food availability [9].

## Supporting Information

Figure S1 **Phylogenetic relationships between the mtDNA haplotypes found in this study (H1–H13; marked with red points) and other published mtDNA control region sequences (N = 243) of European and Siberian roe deer** [5]–[14] **with length of 610 bp.** Numbers at nodes show support (≥50%) from 10.000 bootstrap replicates. European roe deer clades are defined according to Randi *et al*. [5]. Each clade is marked with a different color.
(TIF)

Figure S2 **Phylogenetic relationship between the mtDNA haplotypes found in this study (H1–H13; marked with red points) and other published mtDNA control region sequences (N = 215) of European and Siberian roe deer** [5]–[13] **with length of 510 bp.** Numbers at nodes show support (≥50%) from 10.000 bootstrap replicates. European roe deer clades are defined according to Randi *et al*. [5]. Each clade is marked with a different color. Due to the shortening of our sequences, there are no differences between haplotypes H2 and H13.
(TIF)

Table S1 **Microsatellite diversity of roe deer at three sampling areas in northeastern Poland.** N = number of samples, A = number of alleles, He = expected heterozygosity, Ho = observed heterozygosity.
(DOCX)

References S1 **References for supporting information.**
(DOCX)
