# Supplementary figures and images for: Weak Population Structure in European Roe Deer (Capreolus capreolus) and Evidence of Introgressive Hybridization with Siberian Roe Deer (C. pygargus) in Northeastern Poland
Source: PLoS One. 2014 Oct 1;9(10):e109147. doi: 10.1371/journal.pone.0109147 (PMC4182808; doi:10.1371/journal.pone.0109147)

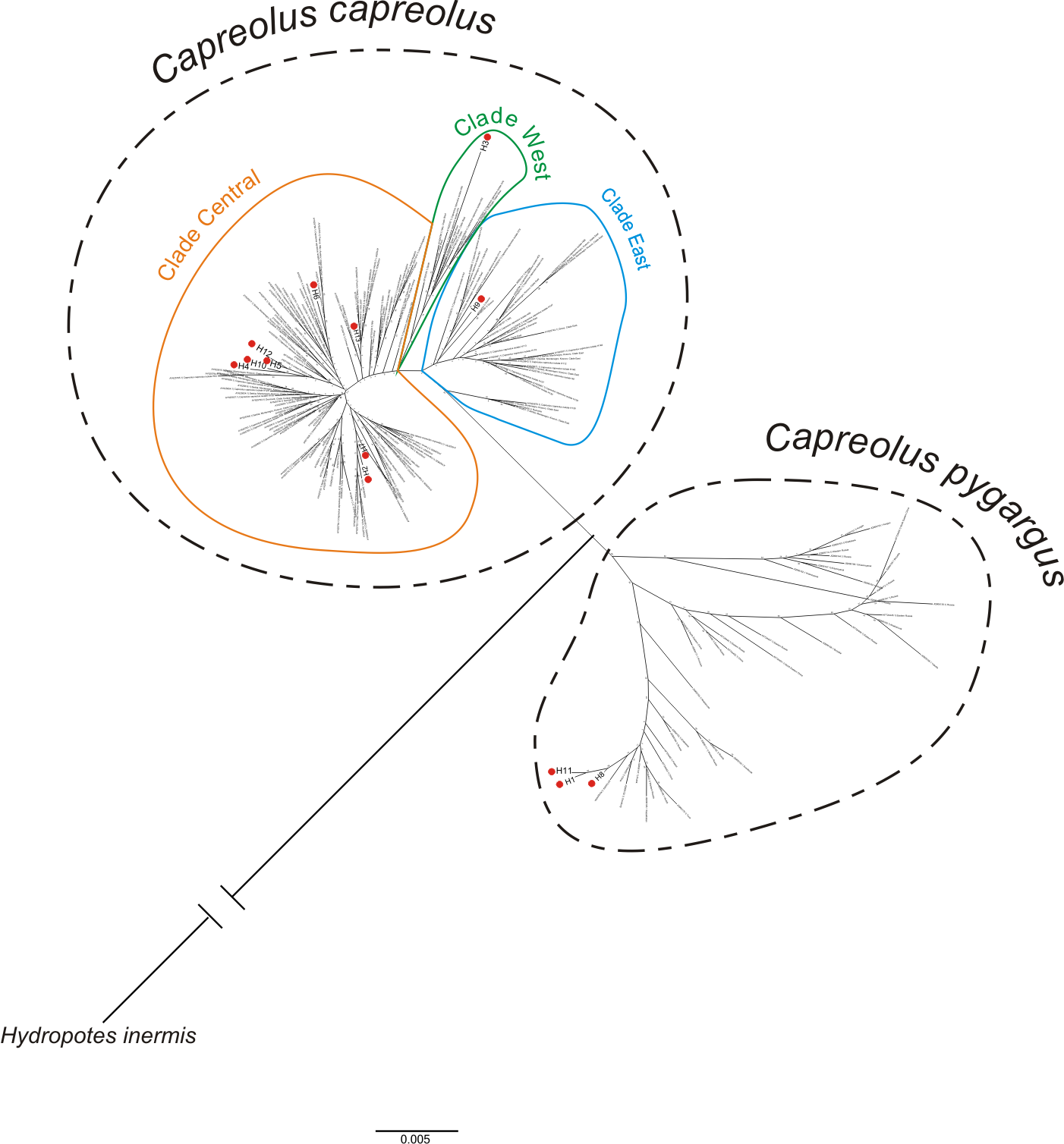

Supplement: Figure S1 — Phylogenetic relationships between the mtDNA haplotypes found in this study (H1–H13; marked with red points) and other published mtDNA control region sequences (N = 243) of European and Siberian roe deer [5]–[14] with length of 610 bp. Numbers at nodes show support (≥50%) from 10.000 bootstrap replicates. European roe deer clades are defined according to Randi et al. [5]. Each clade is marked with a different color. (TIF) [file pone.0109147.s001.tif]

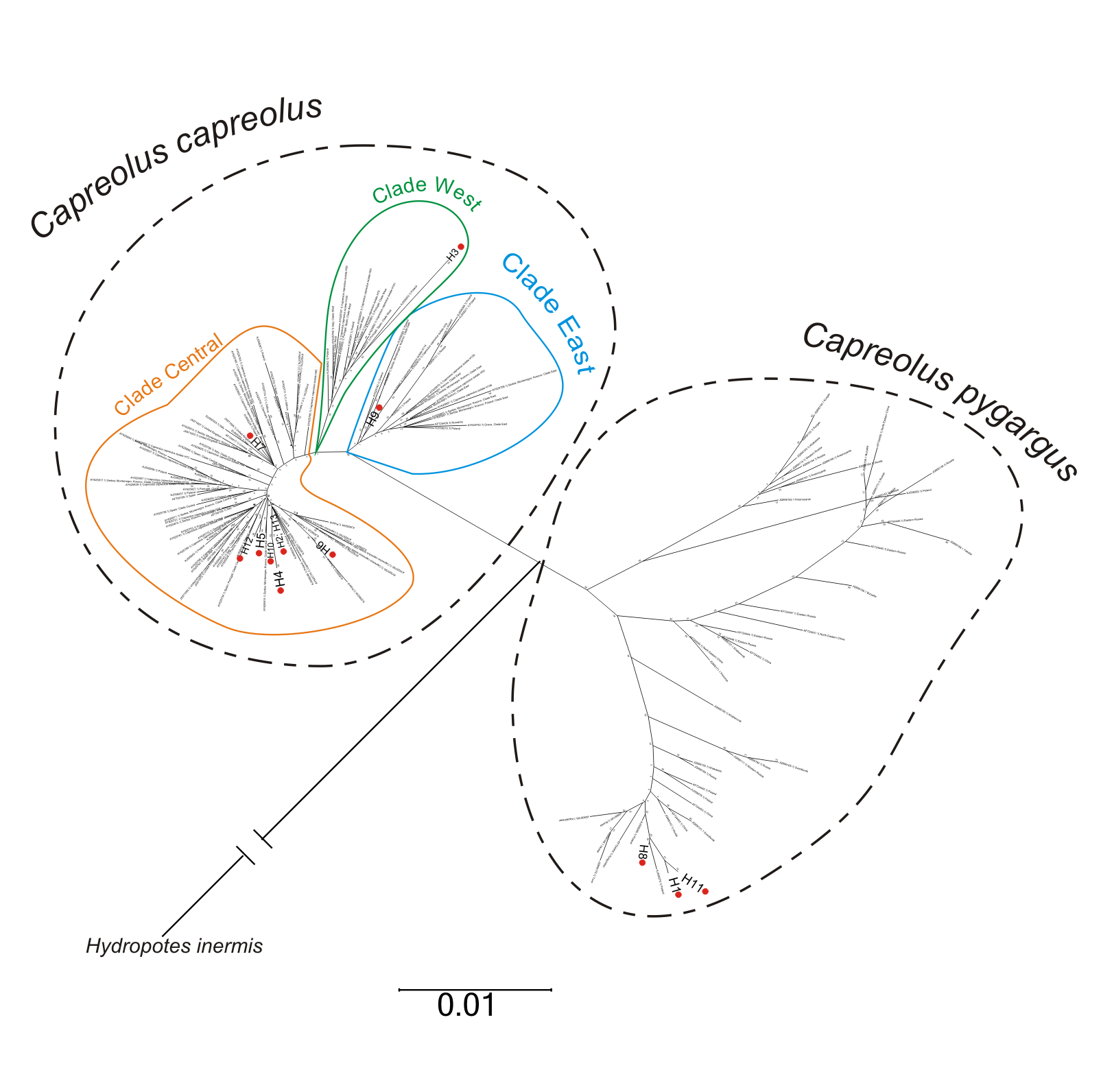

Supplement: Figure S2 — Phylogenetic relationship between the mtDNA haplotypes found in this study (H1–H13; marked with red points) and other published mtDNA control region sequences (N = 215) of European and Siberian roe deer [5]–[13] with length of 510 bp. Numbers at nodes show support (≥50%) from 10.000 bootstrap replicates. European roe deer clades are defined according to Randi et al. [5]. Each clade is marked with a different color. Due to the shortening of our sequences, there are no differences between haplotypes H2 and H13. (TIF) [file pone.0109147.s002.tif]
