# Supplementary material for: Weak Population Structure in European Roe Deer (Capreolus capreolus) and Evidence of Introgressive Hybridization with Siberian Roe Deer (C. pygargus) in Northeastern Poland
Source: PLoS One. 2014 Oct 1;9(10):e109147. doi: 10.1371/journal.pone.0109147 (PMC4182808; doi:10.1371/journal.pone.0109147)
Supplement: Table S1 — Microsatellite diversity of roe deer at three sampling areas in northeastern Poland. N = number of samples, A = number of alleles, He = expected heterozygosity, Ho = observed heterozygosity. (DOCX) [file pone.0109147.s003.docx]

**Table S1.** Microsatellite diversity of roe deer at three sampling areas in northeastern Poland. N = number of samples, A = number of alleles, He = expected heterozygosity, Ho = observed heterozygosity.

| Locus | Reference^a^ | Multiplex^b^ | Allele size range (bp) |  | | | | Sampling site | | | |  | | | |
| --- | --- | --- | --- | --- | --- | --- | --- | --- | --- | --- | --- | --- | --- | --- | --- |
|  |  |  |  | Białowieża (N = 230) | | | | Knyszyn (N = 20) | | | | Augustów (N = 69) | | | |
|  |  |  |  | A | He | Ho | Fis | A | He | Ho | Fis | A | He | Ho | Fis |
| BM1818 | [1] | 1^c^ | 247-263 | 7 | 0.70 | 0.64 | 0.08 | 7 | 0.71 | 0.65 | 0.09 | 6 | 0.72 | 0.63 | 0.12 |
| BM757 | [1] | 1^c^ | 158-211 | 14 | 0.80 | 0.82 | -0.03 | 8 | 0.72 | 0.75 | -0.04 | 13 | 0.84 | 0.91 | -0.09 |
| CSSM66 | [2] | 1^c^ | 167-185 | 6 | 0.71 | 0.71 | 0.00 | 4 | 0.68 | 0.70 | -0.03 | 5 | 0.63 | 0.54 | 0.15 |
| NVHRT21 | [3] | SR^d^ (1) | 152-178 | 12 | 0.85 | 0.81* | 0.04 | 9 | 0.79 | 0.85 | -0.08 | 11 | 0.79 | 0.84 | -0.06 |
| NVHRT73 | [3] | 2^e^ | 210-267 | 9 | 0.42 | 0.31* | 0.26 | 5 | 0.32 | 0.30 | 0.06 | 4 | 0.18 | 0.07* | 0.59 |
| NVHRT71 | [3] | 2^e^ | 97-125 | 6 | 0.50 | 0.29* | 0.42 | 3 | 0.53 | 0.00* | 1.00 | 4 | 0.60 | 0.27* | 0.55 |
| NVHRT24 | [3] | 2^e^ | 110-150 | 8 | 0.58 | 0.50 | 0.13 | 6 | 0.63 | 0.55 | 0.13 | 7 | 0.66 | 0.65 | 0.01 |
| NVHRT48 | [3] | 3^f^ | 80-95 | 4 | 0.54 | 0.54 | 0.00 | 4 | 0.51 | 0.60 | -0.18 | 4 | 0.49 | 0.52 | -0.06 |
| NVHRT16 | [3] | 3^f^ | 151-175 | 10 | 0.77 | 0.75 | 0.03 | 7 | 0.72 | 0.70 | 0.03 | 9 | 0.78 | 0.75 | 0.03 |
| RT1 | [4] | 3^f^ | 210-245 | 13 | 0.88 | 0.82 | 0.07 | 8 | 0.87 | 0.75 | 0.14 | 10 | 0.88 | 0.83 | 0.06 |
| BMS119 | [1] | SR^g^ (3) | 110 | 1 | 0.00 | 0.00 | - | 1 | 0.00 | 0.00 | - | 1 | 0.00 | 0.00 | - |
| MCM64 | [2] | SR^h^ (3) | 123-153 | 8 | 0.65 | 0.63 | 0.03 | 6 | 0.55 | 0.60 | -0.09 | 6 | 0.58 | 0.56 | 0.03 |
| Roe1 | [1] | 4^i^ | 131-133 | 2 | 0.43 | 0.58* | -0.36 | 2 | 0.30 | 0.35 | -0.19 | 2 | 0.39 | 0.49 | -0.27 |
| ETH225 | [1] | 4^i^ | 137-155 | 7 | 0.75 | 0.51* | 0.33 | 6 | 0.74 | 0.45* | 0.40 | 6 | 0.72 | 0.51* | 0.29 |
| Roe8 | [1] | 4^i^ | 59-101 | 10 | 0.77 | 0.77 | 0.01 | 7 | 0.78 | 0.90 | -0.15 | 9 | 0.74 | 0.81 | -0.10 |
| MAF70 | [1] | SR^j^ (4) | 117-155 | 9 | 0.61 | 0.60 | 0.01 | 6 | 0.55 | 0.60 | -0.09 | 10 | 0.68 | 0.72 | -0.06 |

^a^ Original publication where the marker's polymorphism was assessed for roe deer

^b^ Number indicates the multiplex reaction, in which the marker was included; SR indicates a single reaction, followed by the multiplex (in parenthesis) with which it was pooled for running in the sequencer. All reactions were performed with HotStarTaq® Master Mix Kit (Qiagen) in a final volume of 5μl.

^c^ Cycling conditions: 95°C for 15 min / 2 cycles of 94°C for 2 min, 54°C for 20 s, and 72°C for 25 s / 31 cycles of 94°C for 15 s, 54°C for 20 s, and 72°C for 25 s / 72°C for 30 min.

^d^ Cycling conditions: 95°C for 15 min / 30 cycles of 95°C for 1 min, 57°C for 30 s, and 72°C for 1 min / 72°C for 10 min.

^e^ Cycling conditions: 95°C for 15 min / 35 cycles of 95°C for 1 min, 55°C for 30 s, and 72°C for 1 min / 72°C for 10 min.

^f^ Cycling conditions: 95°C for 15 min / 30 cycles of 95°C for 1 min, 55°C for 1 min, and 72°C for 2 min / 72°C for 10 min.

^g^ Cycling conditions: 95°C for 15 min / 10 cycles of 95°C for 30 s, 61°C (decreasing 1°C per cycle) for 30 s, and 72°C for 1 min / 27 cycles of 95°C for 30 s, 51°C for 30 s, and 72°C for 1 min / 72°C for 10 min.

^h^ Cycling conditions: 95°C for 15 min / 32 cycles of 95°C for 1 min, 58°C for 30 s, and 72°C for 1 min / 72°C for 10 min.

^i^ Cycling conditions: 95°C for 15 min / 10 cycles of 95°C for 30 s, 55°C (decreasing 1°C per cycle) for 30 s, and 72°C for 1 min / 17 cycles of 95°C for 30 s, 45°C for 30 s, and 72°C for 1 min / 72°C for 10 min.

^j^ Cycling conditions: 95°C for 15 min / 10 cycles of 95°C for 30 s, 61°C (decreasing 1°C per cycle) for 30 s, and 72°C for 1 min / 20 cycles of 95°C for 30 s, 51°C for 30 s, and 72°C for 1 min / 72°C for 10 min.

* Significant deviation (<0.05) from Hardy-Weinberg equilibrium after false discovery rate correction.
