# Supplementary material for: Weak Population Structure in European Roe Deer (Capreolus capreolus) and Evidence of Introgressive Hybridization with Siberian Roe Deer (C. pygargus) in Northeastern Poland
Source: PLoS One. 2014 Oct 1;9(10):e109147. doi: 10.1371/journal.pone.0109147 (PMC4182808; doi:10.1371/journal.pone.0109147)
Supplement: References S1 — References for supporting information. (DOCX) [file pone.0109147.s004.docx]

**References for Supporting Information**

1. Vial L, Maudet C, Luikart G (2003) Thirty-four polymorphic microsatellites for European roe deer. Mol Ecol Notes 3: 523–527.

2. Røed KH (1998) Microsatellite variation in Scandinavian Cervidae using primers derived from Bovidae. Hereditas 129: 19–25.

3. Røed KH, Midthjell L (1998) Microsatellites in reindeer, *Rangifer tarandus*, and their use in other cervids. Mol Ecol 7: 1773–1776.

4. Poetsch M, Seefeldt S, Maschke M, Lignitz E (2001) Analysis of microsatellite polymorphism in red deer, roe deer, and fallow deer - possible employment in forensic applications. Forensic Sci Int 116: 1–8.

5. Randi E, Alves P, Carranza J, Milosevic-Zlatanovic S, Sfougaris A, *et al.* (2004) Phylogeography of roe deer (*Capreolus capreolus*) populations: the effects of historical genetic subdivisions and recent nonequilibrium dynamics. Mol Ecol 13: 3071–3083.

6. Xiao C-T, Zhang M-H, Fu Y, Koh H-S (2007) Mitochondrial DNA distinction of northeastern China roe deer, Siberian roe deer, and European roe deer, to clarify the taxonomic status of northeastern China roe deer. Biochem Genet 45: 93–102.

7. Gentile G, Vernesi C, Vicario S, Pecchioli E, Caccone A, *et al.* (2009) Mitochondrial DNA variation in roe deer (*Capreolus capreolus*) from Italy: evidence of admixture in one of the last *C. c. italicus* pure populations from central-southern Italy. Ital J Zool 76: 16–27.

8. Vorobieva NV, Sherbakov DY, Druzhkova AS, Stanyon R, Tsybankov AA, *et al.* (2011) Genotyping of *Capreolus pygargus* fossil DNA from Denisova cave reveals phylogenetic relationships between ancient and modern populations. PLoS ONE 6(8): e24045.

9. Zvychainaya EY, Danilkin AA, Kholodova MV, Sipko TP, Berber AP (2011) Analysis of the variability of the control region and cytochrome b gene of mtDNA of *Capreolus pygargus* Pall. Biol Bull 38: 434–439.

10. Zvychaynaya EY, Kiryakulov VM, Kholodova MV, Danilkin AA (2011) Roe deer (*Capreolus*) from Moscow area: analysis of mitochondrial control region polymorphism. Vestn Okhotovedeniya 8: 168–172.

11. Baker KH, Hoelzel AR (2012) Evolution of population genetic structure of the British roe deer by natural and anthropogenic processes (*Capreolus capreolus*). Ecol Evol 3: 89–102.

12. Koh HS, Bayarlkhagva D, Jang KH, Han ED, Jo JE, *et al.* (2013) Genetic divergence of the Siberian roe deer from Korean Jeju Island (*Capreolus pygargus ochraceus*), reexamined from nuclear IRBP and mitochondrial cytochrome b and control region sequences of *C. pygargus*. J Biol Res 19: 46–55.

13. Lorenzini R, Garofalo L, Qin X, Voloshina I, Lovari S (2014) Global phylogeography of the genus *Capreolus* (Artiodactyla: Cervidae), a Palaearctic meso-mammal. Zool J Linn Soc 170: 209–221.

14. Matosiuk M, Borkowska A, Świsłocka M, Mirski P, Borowski Z, *et al.* (2014) Unexpected population genetic structure of European roe deer in Poland: an invasion of the mtDNA genome from Siberian roe deer. Mol Ecol 23: 2559–2572.
